# Supplementary figures and images for: Latent class analysis of barriers to HIV testing services and associations with sexual behaviour and HIV status among adolescents and young adults in Nigeria
Source: PLoS One. 2024 Apr 18;19(4):e0300220. doi: 10.1371/journal.pone.0300220 (PMC11025812; doi:10.1371/journal.pone.0300220)

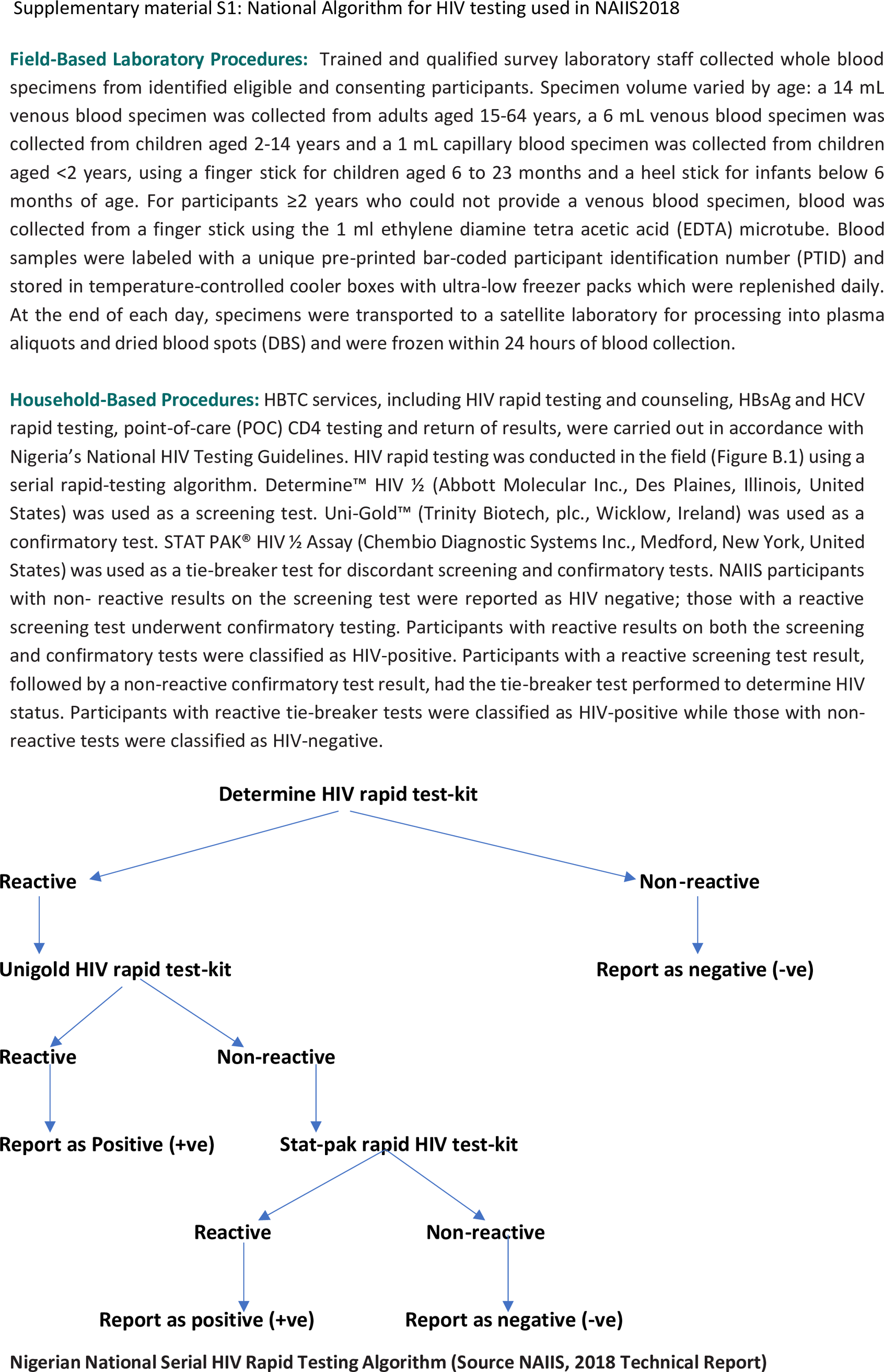

Supplement: S1 File — (TIF) [file pone.0300220.s001.tif]

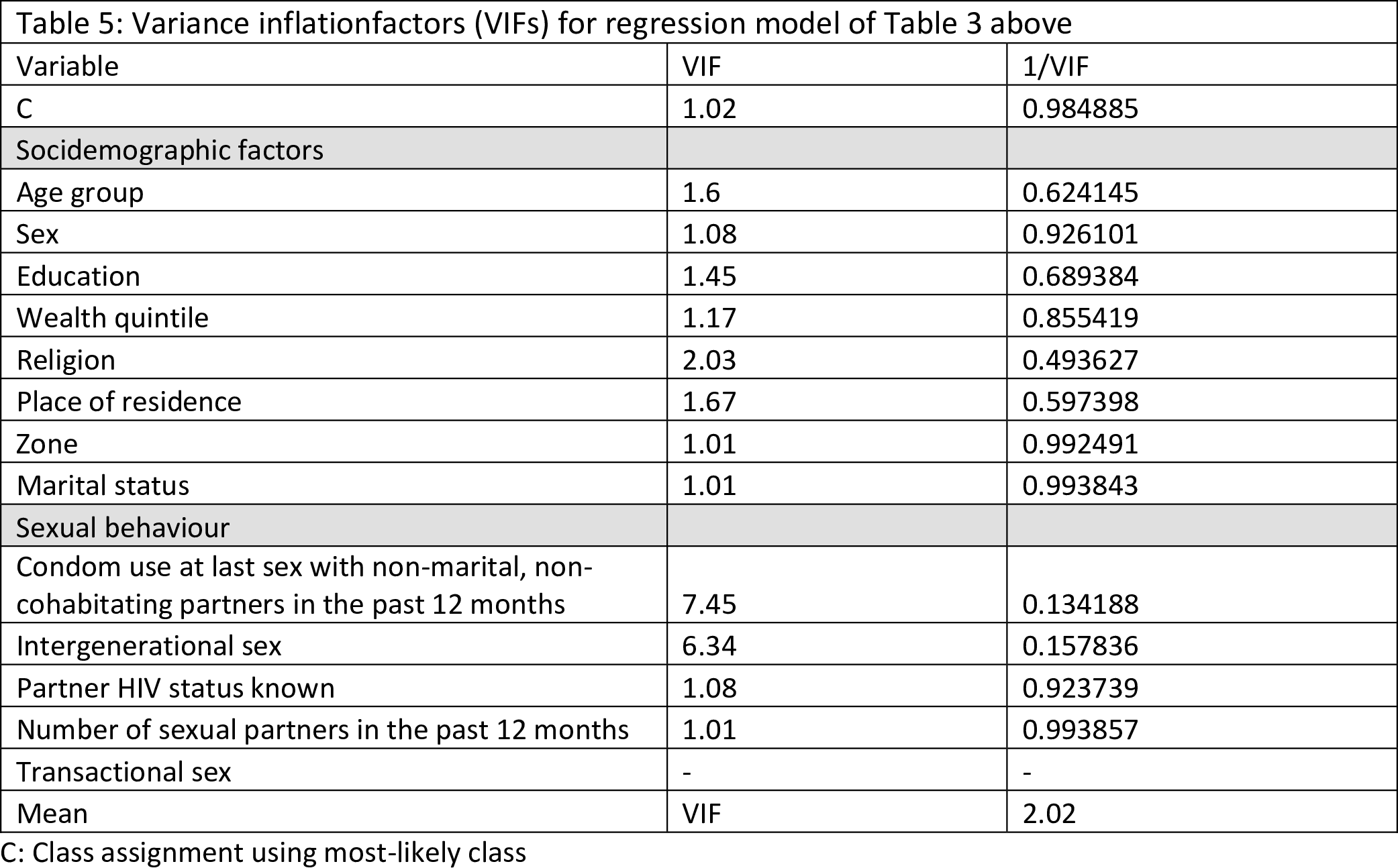

Supplement: S2 File — (TIF) [file pone.0300220.s002.tif]

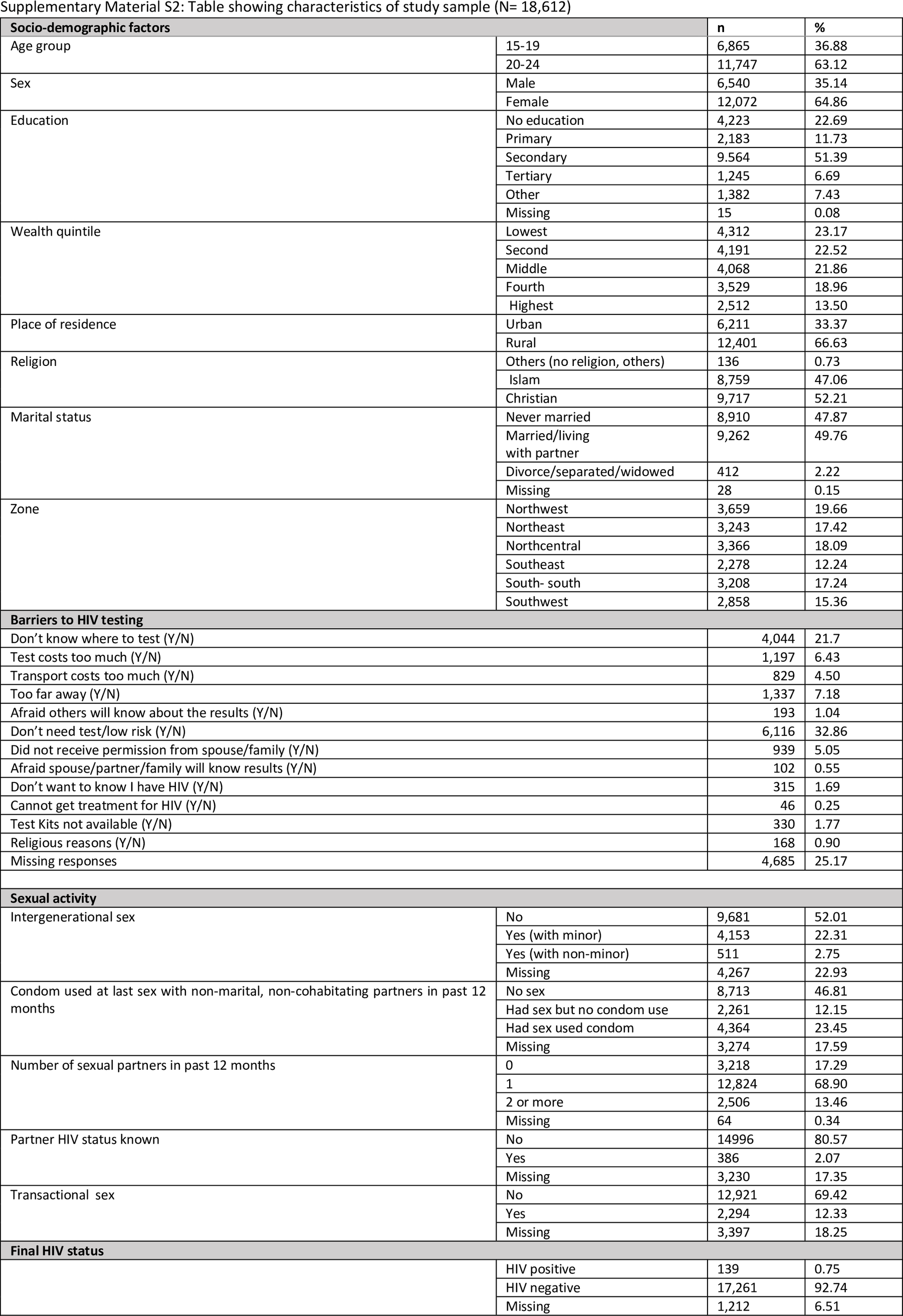

Supplement: S1 Table — (TIF) [file pone.0300220.s003.tif]

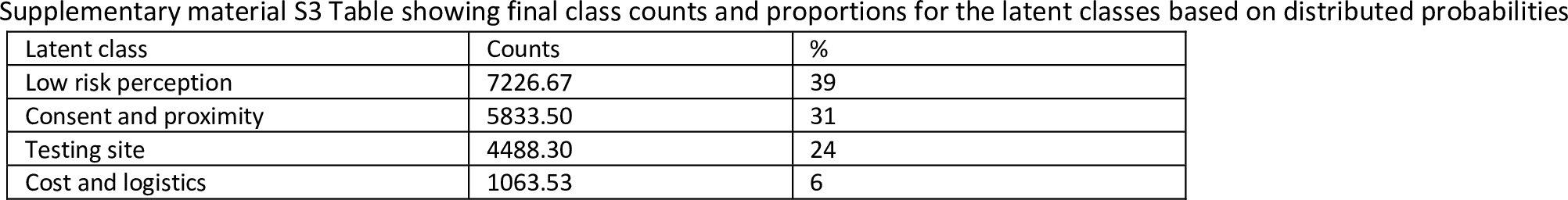

Supplement: S2 Table — (TIF) [file pone.0300220.s004.tif]

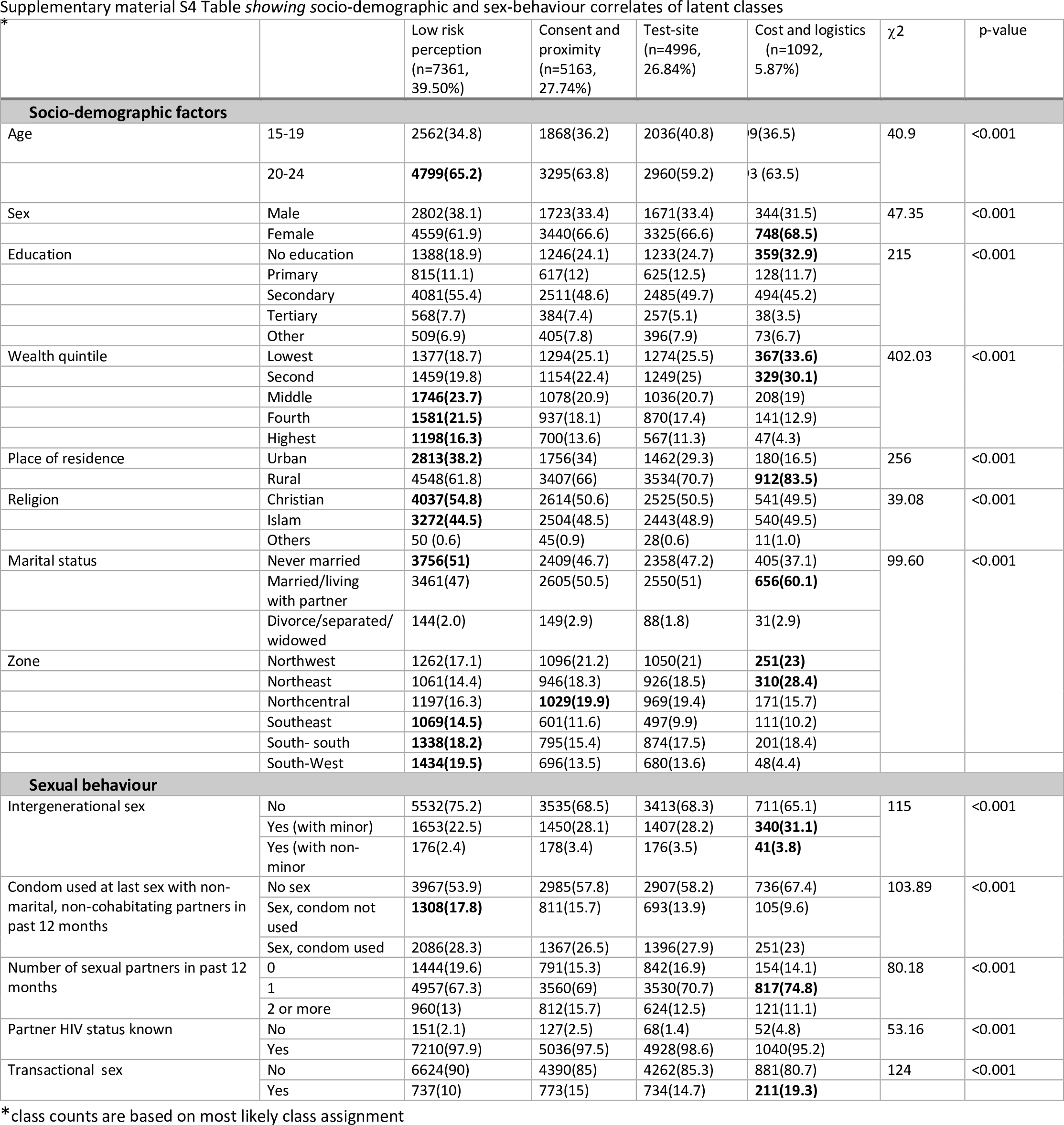

Supplement: S3 Table — (TIF) [file pone.0300220.s005.tif]

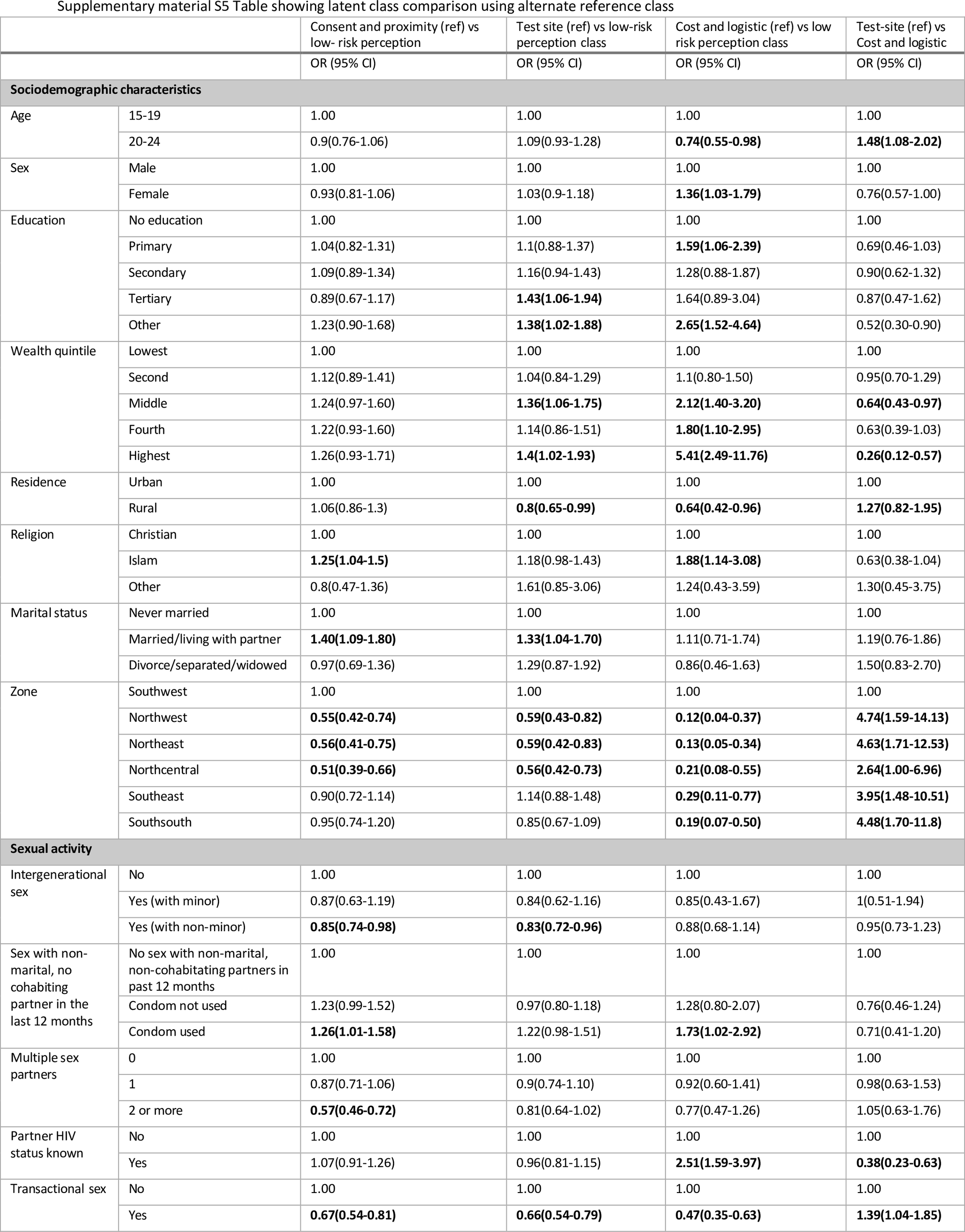

Supplement: S4 Table — (TIF) [file pone.0300220.s006.tif]
