# Supplementary material for: Latent class analysis of barriers to HIV testing services and associations with sexual behaviour and HIV status among adolescents and young adults in Nigeria
Source: PLoS One. 2024 Apr 18;19(4):e0300220. doi: 10.1371/journal.pone.0300220 (PMC11025812; doi:10.1371/journal.pone.0300220)
Supplement: Attachment — Submitted filename: Intersecting HIV testing barriers.pdf [file pone.0300220.s007.pdf]

Intersecting HIV testing barriers, sexual behaviour and undiagnosed HIV: a latent class approach to understanding HIV vulnerability among adolescents and young adults in Nigeria

This paper addresses a pertinent issue by examining the obstacles to HIV testing among a group that has received limited attention in research—adolescents and young individuals. By employing latent class analysis, multinomial regression, and binary logistic regression, the study offers significant findings regarding the correlates of HIV testing barriers and HIV infection. Nevertheless, the paper is not reader-friendly and major revisions are necessary for this paper prior to publications.

General comments

1. This paper contains numerous typos and grammatical errors that require correction before publication.

Specific comments

Title

2. The paper's title does not accurately represent its content. While this study does not primarily focus on the intersectionality of barriers to HIV testing, its main objective is to determine the factors or correlates associated with these barriers. To achieve this, the researchers employed LCA to condense the 12-item questionnaire on HIV testing barriers into four latent classes. These classes were then combined into a variable that served as the primary outcome, although this was not explicitly stated. It is important to note that the utilization of LCA in this study aimed to identify four distinct groups of individuals with similar response patterns to the questions regarding HIV testing barriers. The correlates of HIV were not assessed by LCA as stated in the title but with binary logistic regression. Furthermore, no analysis was conducted to demonstrate the co-occurrence of HIV testing barriers, sexual behavior, and undiagnosed HIV.
3. There is nothing in the paper that indicated “undiagnosed HIV”. This is a complex concept to measure.

Abstract

4. Page 1, lines 35-39: The introduction was incongruous with the rest of the paper.
5. Page 1, lines 37-39” *“….and how these intersections impact patterns of sexual behavior and the prevalence of undiagnosed HIV among adolescents and young people in Nigeria*.”: The study did not provide any specific evidence on how the intersections influenced sexual behavior or the prevalence of undiagnosed HIV among adolescents and young people in Nigeria. Additionally, factors related to sexual behavior were considered as potential confounding variables in the association between the latent classes of HIV testing barriers (primary outcome variable) and sociodemographic variables (independent factors). The objective of the study is unclear and needs to be rephrased.
6. Page 1, lines 40-43: The authors should clearly specify the methods employed to tackle both the primary and secondary objectives, as well as define the primary and secondary outcome variables. Based on the main findings, it seems that the authors utilized latent clusters as the primary outcome measure, and HIV status as the secondary outcome variable.
7. Page 1, line 43: The term "*HIV prevalence*" is misleading in this context. It appears that the authors were actually referring to the HIV status determined from the blood samples collected.

8. Page 1, line 44: I would suggest renaming the clusters to improve clarity. Here are my suggestions: Cluster 1: Low-risk perception cluster, cluster 2: Consent and proximity cluster, cluster 3: Testing site cluster and cluster 4: Cost and logistics cluster.
9. Page 1, line 46: Regarding HIV, it is important to clarify that the percentage of positive test results represents the HIV positivity rate, rather than prevalence. The term 'prevalence' encompasses a broader scope of HIV infection within a particular population." Recall that the study was conducted among AYA who had never been tested for HIV in Nigeria, so prevalence is not applicable.
10. Page 1, line 53: The conclusion does not correspond with the findings.

Introduction:

11. Page 2: The introduction is notably deficient in summarizing the existing body of literature regarding the identifiable factors that impact desired outcomes, particularly in relation to the barriers affecting HIV testing and HIV susceptibility among adolescents and young individuals.
12. Page 3, line 116: The second question asked about the influence of barriers to HIV testing on sociodemographic characteristics and sexual behaviors. However, the intended question should have been about the impact of sociodemographic characteristics and sexual behaviors on different clusters of barriers to HIV testing. Here is the revised question: "What sociodemographic and sexual behaviors influence different clusters of barriers to HIV testing?"
13. Page 3, line 118: The third question appears to be unusual. It assessed the relationship between the clusters of barriers to testing and “undiagnosed HIV”. How can one determine undiagnosed HIV? In the methods section, it was mentioned that the participants had never undergone HIV testing. This suggests that individuals who tested positive contributed to the overall HIV positivity rate. Since the analysis is conducted at the individual level, it would be more appropriate to refer the outcome as HIV-positive.

Methods

14. Page 3, line 124: “Despite economic growth in recent decades, the country continues to face high poverty rates.” What is the method used to measure the poverty rate? Please provide a reference for the regional poverty rates.
15. Page 3, line 154: “According to the national algorithm”: The national algorithm should be stated.
16. Page 4, lines 164-194: It is challenging to determine which variables served as the independent and dependent variables. In contrast to what was stated as the outcome measure in line 194, the primary outcome measure was the barrier to HIV testing, while the secondary outcome measure was HIV status. It is important to state that barrier to HIV testing was selected as an independent variable for the secondary outcome analysis.
17. There is redundancy from lines 170-194 because the same information was presented in Table 1.
18. Table 1 should be summarized for clarity’s sake.
19. Table 1: The authors should note that gender and sex are not the same. While sex is a biological construct (male and female), gender is a social construct. Based on the question asked, sex is more appropriate.
20. Page 6, lines 202-238: Describe the statistical approaches in relation to the objectives. In addition, specify upfront that LCA was employed to reduce data dimensionality of barriers to HIV testing, as its mention arrived quite late in line 220. Also, what type of regression model was performed for HIV status?

Results

21. Table 2 is not clear. Which estimates are frequencies (small n) and medians?
22. How is final HIV status in Table 2 operationalized? Also, why is final HIV status placed under sexual activity?
23. Page 10, line 315 (Table 5): The value of p=0.000 does not exist. When the p-value is extremely low, statistical software approximates it to zero. In such cases, it is more appropriate to report the p-value as p<0.001.
24. Table 5: What is the reason behind certain cells being displayed in bold font?
25. Page 12, line 331: Multinomial regression can not determine association between latent classes. It is used to determine association between independent variables and outcome variable with more than two categories.
26. Page 13: Table 6: Please indicate what the reported magnitude of effect represents. Are they relative risk ratios (RRR) with 95%CI?
27. Page 14, line 373: *"(OR 1.68; CI: 1.04-2.71, p =0.04)"*: State that this is 95%CI.
28. Page 14, line 374: "The association between latent classes and HIV prevalence disappeared". As stated previously, HIV positivity is more appropriate.
29. Table 7 should reflect crude or unadjusted model. The adjusted model is more important to the reader than Table 7 (unadjusted model).

Discussion

30. The identified factors influencing the barriers to HIV testing and HIV infection were not substantially discussed.
31. The spatial pattern of clusters of barriers to HIV testing was completed omitted in the discussion section.
32. Page 16, line 447: "*The complete profiles (HIV prevalence, socio-demographic factors, and sexual behavior) of the four classes, taken together, offer an understanding of potential pathways through which AYA can transition along the spectrum of sexual risk and HIV vulnerability*": This statement is not accurate because pathways can be identified using LCA. If the authors are interested in identifying pathways, they need to run mediation or structural equation models.
