# Supplementary material for: Latent class analysis of barriers to HIV testing services and associations with sexual behaviour and HIV status among adolescents and young adults in Nigeria
Source: PLoS One. 2024 Apr 18;19(4):e0300220. doi: 10.1371/journal.pone.0300220 (PMC11025812; doi:10.1371/journal.pone.0300220)
Supplement: Attachment — Submitted filename: Response to reviewers.docx [file pone.0300220.s008.docx]

Dear Dr. Belayneh Mengist,

Editor,

PLOS ONE

15 October 2023

Resubmission of manuscript “*Latent class analysis of barriers to HIV testing services and associations with sexual behaviour and HIV status among adolescents and young adults in Nigeria”*

Dear Dr Mengist,

We are pleased to submit our revised manuscript entitled: “*Latent class analysis of barriers to HIV testing services and associations with sexual behaviour and HIV status among adolescents and young adults in Nigeria”* to the PLOS ONE for consideration for publication. We appreciate the careful review and constructive suggestions.

We believe that the manuscript is substantially improved after addressing editor and reviewers’ comments. Submitted with this letter are the editor and reviewers’ comments with our responses in tabular form, including how and where the text was modified.

The revision has been developed in consultation with all co-authors, and each author has seen and approved the final form of this revision. The funders had no role in study design, data collection and analysis, decision to publish, or preparation of the manuscript.

Thank you for your consideration of our revised paper.

Sincerely,

Okikiolu Badejo for the co-authors

|  | Editor’s comment | Authors’ response | Location in revised manuscript |
| --- | --- | --- | --- |
| 1 | 1. Please ensure that your manuscript meets PLOS ONE's style requirements, including those for file naming. The PLOS ONE style templates can be found at  <https://journals.plos.org/plosone/s/file?id=wjVg/PLOSOne_formatting_sample_main_body.pdf> and  <https://journals.plos.org/plosone/s/file?id=ba62/PLOSOne_formatting_sample_title_authors_affiliations.pdf> | We thank the editor for their constructive reviews. We have now aligned the manuscript with PLOS ONE style requirements |  |
| 2 | 2. Thank you for stating the following financial disclosure:  “OB was supported with research funding from the Belgium Directorate-General for Development Cooperation (DGD) awarded through the Institute of Tropical Medicine Antwerp, Belgium. SVB received funding from the Flanders Research Foundation (FWO) grant number 1221821N.”  Please state what role the funders took in the study.  If the funders had no role, please state: "The funders had no role in study design, data collection and analysis, decision to publish, or preparation of the manuscript."  If this statement is not correct you must amend it as needed.  Please include this amended Role of Funder statement in your cover letter; we will change the online submission form on your behalf." | Thank you. We have now included this statement "The funders had no role in study design, data collection and analysis, decision to publish, or preparation of the manuscript” in the Funding statement of the manuscript and included it in our cover letter. | Line 545-546 |
| 3 | 3. In your Data Availability statement, you have not specified where the minimal data set underlying the results described in your manuscript can be found. PLOS defines a study's minimal data set as the underlying data used to reach the conclusions drawn in the manuscript and any additional data required to replicate the reported study findings in their entirety. All PLOS journals require that the minimal data set be made fully available. For more information about our data policy, please see <http://journals.plos.org/plosone/s/data-availability>.  "Upon re-submitting your revised manuscript, please upload your study’s minimal underlying data set as either Supporting Information files or to a stable, public repository and include the relevant URLs, DOIs, or accession numbers within your revised cover letter. For a list of acceptable repositories, please see <http://journals.plos.org/plosone/s/data-availability#loc-recommended-repositories>. Any potentially identifying patient information must be fully anonymized.  Important: If there are ethical or legal restrictions to sharing your data publicly, please explain these restrictions in detail. Please see our guidelines for more information on what we consider unacceptable restrictions to publicly sharing data: <http://journals.plos.org/plosone/s/data-availability#loc-unacceptable-data-access-restrictions>. Note that it is not acceptable for the authors to be the sole named individuals responsible for ensuring data access. We will update your Data Availability statement to reflect the information you provide in your cover letter. | We thank the editor for the comment. As per PLOS ONE requirement, we have included the following details in the data availability statement during submission:   1. A description of the data set and the third-party source 2. If applicable, verification of permission to use the data set 3. All necessary contact information others would need to apply to gain access to the data |  |
| 4 | 4. We note that Figure 2 in your submission contain [map/satellite] images which may be copyrighted. All PLOS content is published under the Creative Commons Attribution License (CC BY 4.0), which means that the manuscript, images, and Supporting Information files will be freely available online, and any third party is permitted to access, download, copy, distribute, and use these materials in any way, even commercially, with proper attribution. For these reasons, we cannot publish previously copyrighted maps or satellite images created using proprietary data, such as Google software (Google Maps, Street View, and Earth). For more information, see our copyright guidelines: <http://journals.plos.org/plosone/s/licenses-and-copyright>.  We require you to either (1) present written permission from the copyright holder to publish these figures specifically under the CC BY 4.0 license, or (2) remove the figures from your submission:  1. You may seek permission from the original copyright holder of Figure 2 to publish the content specifically under the CC BY 4.0 license.  We recommend that you contact the original copyright holder with the Content Permission Form (<http://journals.plos.org/plosone/s/file?id=7c09/content-permission-form.pdf>) and the following text:  “I request permission for the open-access journal PLOS ONE to publish XXX under the Creative Commons Attribution License (CCAL) CC BY 4.0 (<http://creativecommons.org/licenses/by/4.0/>). Please be aware that this license allows unrestricted use and distribution, even commercially, by third parties. Please reply and provide explicit written permission to publish XXX under a CC BY license and complete the attached form.”  Please upload the completed Content Permission Form or other proof of granted permissions as an "Other" file with your submission.  In the figure caption of the copyrighted figure, please include the following text: “Reprinted from [ref] under a CC BY license, with permission from [name of publisher], original copyright [original copyright year].”  2. If you are unable to obtain permission from the original copyright holder to publish these figures under the CC BY 4.0 license or if the copyright holder’s requirements are incompatible with the CC BY 4.0 license, please either i) remove the figure or ii) supply a replacement figure that complies with the CC BY 4.0 license. Please check copyright information on all replacement figures and update the figure caption with source information. If applicable, please specify in the figure caption text when a figure is similar but not identical to the original image and is therefore for illustrative purposes only.  The following resources for replacing copyrighted map figures may be helpful:  USGS National Map Viewer (public domain): <http://viewer.nationalmap.gov/viewer/>  The Gateway to Astronaut Photography of Earth (public domain): <http://eol.jsc.nasa.gov/sseop/clickmap/>  Maps at the CIA (public domain): <https://www.cia.gov/library/publications/the-world-factbook/index.html> and <https://www.cia.gov/library/publications/cia-maps-publications/index.html>  NASA Earth Observatory (public domain): <http://earthobservatory.nasa.gov/>  Landsat: <http://landsat.visibleearth.nasa.gov/>  USGS EROS (Earth Resources Observatory and Science (EROS) Center) (public domain): [http://eros.usgs.gov/#](http://eros.usgs.gov/)  Natural Earth (public domain): <http://www.naturalearthdata.com/> | Thank you. All maps in the study were created exclusively by the authors using map data that are publicly available. As per PLOS ONE requirement we have now located and cited the source of the base map data in line with the copyright requirements for attribution | Lines 161 and 368 |
| 5 | 5. Please include captions for your Supporting Information files at the end of your manuscript, and update any in-text citations to match accordingly. Please see our Supporting Information guidelines for more information: <http://journals.plos.org/plosone/s/supporting-information>. | We appreciate the editor’s comment. We have now included caption for all supporting information in separate files with appropriate caption and in-text citation. |  |
|  | Reviewer 1 | Authors’ response | Location in revised manuscript |
| 1 | Abstract: You have mentioned how less is known about barriers interacting across multiple levels but have not cited the applicability of LCA to be able to capture this. Would be useful to comment on LCA’s ability to derive homogeneity between different barriers and thus capture their intersection, as well as maybe a brief statement about what LCA is (a mixture-modelling technique deriving a latent variable etc.). | We thank the reviewer for their comment. The limited word count in the abstract does not allow us to do this. We have however included an introduction to LCA and its application to our study in the introduction section, as per your other comments below. |  |
| 2 | Line 43: Is sexual behaviour an actual covariate or is it a theme of covariates? Sounds very broad, would be useful to list the actual covariates used. Needs a full stop at the end of the paragraph | Thank you. We have listed the variables we refer to as sexual behaviour. | Lines 45-47 |
| 3 | Lines 44-45: Please clarify in the abstract that the bracketed values are the proportion of the population that are assigned to each latent class, might not be clear. | This has been done, thank you. | Lines 48-50 |
| 4 | Lines 46-48: Again, would be useful to get an indication of what these sexual practices are that you are measuring when you say riskier sexual behaviours. | This has been done, thank you. | Lines 50-52 |
| 5 | Lines 48-50: Consistency in reporting of HIV prevalence, you have reported proportions for all except test-permission denied, which you have reported an OR. | Thank you. We have rephrased the abstract removing all instances of data on HIV positivity to avoid confusion. |  |
| 6 | Line 51: What sociodemographic factors? Also, confusing about which classes have higher odds of HIV infection, is it just low-risk perception which you have reported the OR for, or is it also test-permission denied and test-permission denied. | Thank you. We have removed all mention of odd ratios and proportion reporting for the classes on HIV positivity to avoid confusion. |  |
| 7 | Line 52: Full stop at end of paragraph. | Thank you. We have done this. | Line 47, 55 |
| 8 | Line 69: Citation for end to AIDS pandemic. | We have done this. |  |
| 9 | Line 82: need citation for period of adolescence and use being characterised by increased sexual risk taking, unless it is the same for the point about risk taking being associated with increased HIV acquisition? | We have rewritten whole sections of the introduction and taken out this section. |  |
| 10 | Line 85: citation needed | We have included citation. | Lines 69-83 |
| 11 | Line 92: citation needed | We have included citation. |  |
| 12 | Line 96: citation needed, even If the same as that in line 99 | We have included citation. |  |
| 13 | Line 97: maybe instead of “according to the UNAIDS 95-95-95 cascade” you could say “Appraising Nigeria’s current HIV landscape against the UNAIDS 95-95-95 cascade” | We have rewritten whole sections of the introduction and taken out this section. |  |
| **14** | **Introduction would benefit** from a methodological introduction to LCA, and how this technique can be used to “account for the heterogeneity within the AYA population” and the “complex relationships between factors at different levels” | We have done this. | Lines 108-128 |
| 15 | Line 107: citation needed | We have rewritten whole sections of the introduction and taken out this section. |  |
| 16 | Line 125: citation needed | We have included citation. | Lines 155-160 |
| 17 | Line 126: citation needed | We have included citation. |  |
| 18 | Lines 138-140: Would be useful to know the response rates of the surveys | We have included this information. | Lines 177-182 |
| 19 | Figure 1: So vaginal sex was used to determine whether people were sexually active? That is an important thing to note in the methods, as it is making an important and perhaps controversial, definition of sexually active. For example, it is likely to be excluding sexual minority groups who do not enact this type of sex, or those who practice primarily non-penetrative sex. | This has been noted in the methods, and we also reflect on the limitations in the section on study limitations. | Lines 210-211, 514-517 |
| 20 | Understand it is probably too late to change this now, however this has to be noted as the definition of sexually active in line 159, and has to be highlighted as a limitation in the discussion, particularly mentioning its potential exclusion of sexual minority groups (who are a key population within the HIV epidemic of Nigeria - <https://bmcpublichealth.biomedcentral.com/articles/10.1186/s12889-019-7540-4> ). Also negates the potential transmission of HIV through oral sex, which is another limitation that should be mentioned. | This has been noted in the methods, and the limitations reflected upon in the section on study limitations. | Lines 210-211; 514-517 |
| 21 | Lines 181-185: Don’t think you need this given that you have already mentioned the variables you have been investigating. | Thank you. This has been removed. |  |
| 22 | Line 194: final HIV status from the blood test? | Details of the algorithm to determine Final HIV status has been included in Table 1 and in text as well as in Supporting Information. | Lines 193-199 |
| 23 | Table 1: fine to recode religion into a binary category as this is often required to perform regression analyses on an LCA model, but the limitations of this in being reductive and potentially missing important religious subgroups/denominations should be mentioned in the discussion. | This has been noted in the section on study limitations. | Lines 523-524 |
| 24 | Table 1: Be consistent in the table with putting ‘Not recoded’, as it is missing from ‘Gender’, for example. Would be interested to see if there were any trans/non-binary patients who did not answer this question, and how their data was handled. If individuals not answering the gender question were excluded, this is a very important limitation to cite. | We have corrected this to ensure consistency in “non recoded”. NAIIS2018 only included two categories (male/female) for sex/gender in its questionnaires. We have noted the potential limitation of excluding other categories in the study limitation section in lines 519-523. | Table 1 (line  213); Lines 519-523 |
| 25 | Table 1: Martial status recode column is the same as that for religion. | Thank you. This has been corrected. | Table 1 (Line 213) |
| 26 | Table 1: Gender question will not capture those who are trans or non-binary. Similarly, if the data does not present this there is nothing you can do, however, it is important to mention this in the discussion, with this again being an important population in the Nigerian HIV epidemic (<https://www.ncbi.nlm.nih.gov/pmc/articles/PMC7906338/>) | As per the second reviewer’s comment, we have changed the wording to “sex” rather than gender as this is more appropriate. However, we have noted the limitation of NAIISS binary categorisation (Male and Female), and the potential impact of exclusion of sexual minority groups on our study findings . | Line 519-523 |
| 27 | Line 198: cite STATA here. | This has been done. | Line 216 |
| 28 | Line 203: cite Mplus here. | Done. | Line 218 |
| 29 | Line 212: You need to state clearly which variables were used as manifest variables to construct the classes, and how these were chosen (i.e., previous research, previous LCA models) | This has now been well clearly stated. | Line 218-221 |
| 30 | Line 209: citation needed. | Done. | Line 228 |
| 31 | Line 210: missing a closed bracket at the end of the sentence | We have corrected this. | Line228 |
| 32 | Line 214-215: don’t think you need the expansion of the maximum probability rule, think the space would be better use explaining what the LCA model is | Thank you, we have removed the redundant section. |  |
| 33 | Line 230: cite this | Done. | Line 252 |
| 34 | Lines 240-254: think this should go in the Ethics statement at the beginning of the paper. | We have followed the journal manuscript template which indicates this to be in text at the end of the methods. |  |
| 35 | Understand if space is limited, but would be interesting to get an idea of the proportions of the sample who reported each barrier to HIV testing (those highlighted in lines 170-177) Line 257: reference figure 1 here, and potentially consider moving this figure to this point in the paper. | This is now included in the table on sample characteristics, now in Supporting Information S2 per other reviewer comment. | Supporting Information S2 |
| 36 | Line 257: ‘Sample characteristics’. When you say overall study, does this mean that this is the demographics of the 18,612 participants in the LCA model, or the overall 229,762 participants in NAIIS? If it is the latter, I think this table should be redone to show the characteristics of only those who are in the LCA model. | We have taken out this table and its descriptive text narrative. The table is now located in Supplementary material S2. | Supporting Information S2 |
| 37 | Line 268: cite this national estimate figure | Done. The table is now located in Supporting Information S2. | Supporting Information S2 |
| 38 | Table 2: “Yes (with non-minor” needs a closing bracket. | Done. The table is now located in Supporting Information S2. |  |
| 39 | Table 2: capitalise ‘missing’ throughout’ | Done. The table is now located in Supporting Information S2. |  |
| 40 | Table 2: incomplete, need to fill in the final HIV status | Done. The table is now located in Supporting Information S2. |  |
| 41 | Table 3: No need to report after 5 classes, up to here is sufficient to show the drop in AIC/BIC which plateaus after 4 class model, and the rising LMR after 4 classes. | Thank you. We have now shown only 6 classes. | Line 321 |
| 42 | Line 282: replace probabilities with “conditional response probabilities”, and this term should be explained in the methods. | We have replaced with conditional response probabilities and explained the term. | Line 237, 323,325,328,332,335341,342 |
| 43 | Table 5: If this the characteristics of the sample in the LCA, then I would remove table 2 and keep this instead. Table 2 is otherwise misleading suggesting this is the demographics of those in the LCA. | We appreciate the reviewer's suggestion. The authors have chosen to relocate the descriptive tables (Tables 2 and 5) to the Supporting Information. This decision aims to retain in the main text only Tables with analytical results that directly address the study objectives. |  |
| 44 | Lines 298-311: include p values for those that are significant | We have done this, thank you. |  |
| 45 | Table 5: need to signify what the bolding means | We appreciate the reviewer's suggestion. We have removed the bolding. In addition, the authors have chosen to relocate the descriptive tables (Tables 2 and 5) to the Supporting Information. This decision aims to retain in the main text only tables with analytical results that directly address the study objectives. | See Supporting Information S4 |
| 46 | Table 5: put χ2 instead of ‘chi2’ in the table | We appreciate the reviewer's suggestion. We have removed the bolding. In addition, the authors have chosen to relocate the descriptive tables (Tables 2 and 5) to the Supporting Information. This decision aims to retain in the main text only tables with analytical results that directly address the study objectives. | See Supporting Information S4 |
| 47 | Line 331: I think instead of association between classes, it should be ‘odds of class assignment’ | We have removed this sub-header to avoid confusion. |  |
| 48 | Line 333-337: In methods section, there needs to be an indication that statistical significance will be assessed by 95% confidence intervals of ORs not overlapping 1 | We thank the reviewer. We have indicated this in lines 258-259. | Line 258-259 |
| 49 | Line 339: remove second comma | Done. |  |
| 50 | Line 343-344: rephrase this, doesn’t read well. | Done. |  |
| 51 | Line 344-345: ‘lower likelihood of knowing partners HIV status’ | Thank you. We have reframed whole section of the result to read better. |  |
| 52 | Table 6: can remove the low risk perception column as this looks messy having a blank table, and put that odds ratios are calculated relative to the low risk perception class in the title of the table. | We appreciate the reviewer’s suggestion and have done removed included a note in the Table title to show the reference category | Current Table 4, line 393 |
| 53 | Table 7 and figure 3 are overlapping on my copy of the manuscript, making Table 7 difficult to read. | We have adjusted the formatting and separated the figure and table. | See line 404, 405 |
| 54 | It is a significant finding that the association between class membership and HIV infection disappeared, so I think that appendix table 3 should be integrated into table 7 | We appreciate the reviewer’s comment. We have integrated the tables into the current Table 5. | Now Table 5 on line 405 |
| 55 | Line 382-384: It is important to acknowledge that this association disappeared when accounting for sociodemographic characteristics and behaviour | Thank you. We have emphasized the disappearance of the effect both in the result and in our discussion, as well as the implications. | Line 398,399, 480-486 |
| 56 | Line 406: LCA acronym can be introduced earlier | Thankyou. We have introduced the acronym earlier in the introduction. | Line 106 |
| 57 | A theme that is not addressed sufficiently in the discussion is that the association of class membership and HIV prevalence disappears when accounting for sociodemographic and risk behaviours, and I think this should be commented upon, as this undermines the importance of barriers in HIV prevalence and implies the importance of sociodemographic characteristics (potentially indicating the role of structural prejudice in informing access) and sexual risk behaviours in driving increased HIV prevalence | We appreciate the reviewer’s comment. We have now highlighted the disappearance of the effect both in the result and in our discussion, as well as the implications. | Line 401-404, 484-489 |
| 58 | Line 466-467: not cited. In general I would recommend going back through the discussion and identifying where there unsupported statements that require some form of citation to support them. | We appreciate the reviewer’s comment. We have included citations for all the relevant statements throughout the manuscript. |  |
| 59 | Line 482: citation needed | We have included relevant citation. | Line 517 |
| 60 | Additional limitations that need to be mentioned: equating of vaginal sex with sexual activity and its subsequent impact on excluding key populations/excluding some potentially risky practices as mentioned above, no analysis on trans/non-binary individuals (even if data did not allow this, the exclusion of this population and its potential impact should be noted as this is a vulnerable population in Nigerian HIV epidemic), | We appreciate the reviewer’s comment. Accordingly, we have included all the stated issues as additional limitations in the appropriate section. | Line 508-523 |
| 61 | methodological limitations of LCA (see other LCA papers in the literature to get an indication of what these are), limitation of most-likely class assignment in not capturing those individuals for whom class assignment was ambiguous (i.e., someone who had a 49% likelihood of being assigned to “logistic constraint” and a 51% likelihood of being assigned to “low-risk perception” is not the same as someone who had a 100% likelihood of being assigned to “low-risk perception”). |  |  |
| 62 | Good manuscript, but needs more citation throughout, more thorough description of the methodology of LCA, its limitations and why it is appropriate for this setting, and the potential key populations that are overlooked in this analysis (i.e., sexual and gender minorities). | We appreciate the reviewer’s comment and constructive reviews which has improved the quality of our work. |  |
|  |  |  |  |
|  | Reviewer 2 | Authors’ response | Location in revised manuscript |
| 1 | This paper addresses a pertinent issue by examining the obstacles to HIV testing among a group that has received limited attention in research—adolescents and young individuals. By employing latent class analysis, multinomial regression, and binary logistic regression, the study offers significant findings regarding the correlates of HIV testing barriers and HIV infection. | We thank the reviewer for their comments on the importance of our work. |  |
| 2 | Nevertheless, the paper is not reader-friendly and major revisions are necessary for this paper prior to publications. | We appreciate the reviewer’s attention to detail and constructive feedback. We have conducted a thorough language and grammar editing, including rewriting substantial sections of the introduction, methods , results, and discussion. |  |
| 3 | This paper contains numerous typos and grammatical errors that require correction before publication. |  |  |
| 4 | Title: The paper's title does not accurately represent its content. While this study does not primarily focus on the intersectionality of barriers to HIV testing, its main objective is to determine the factors or correlates associated with these barriers. To achieve this, the researchers employed LCA to condense the 12-item questionnaire on HIV testing barriers into four latent classes. These classes were then combined into a variable that served as the primary outcome, although this was not explicitly stated. It is important to note that the utilization of LCA in this study aimed to identify four distinct groups of individuals with similar response patterns to the questions regarding HIV testing barriers. The correlates of HIV were not assessed by LCA as stated in the title but with binary logistic regression. Furthermore, no analysis was conducted to demonstrate the co-occurrence of HIV testing barriers, sexual behavior, and undiagnosed HIV. | We have reframed the article’s title to accurately reflect its content. The revised title now reads “Latent class analysis of barriers to HIV testing services and associations with sexual behaviour and HIV status among adolescents and young adults in Nigeria”, which removes any incorrect assumption that the LCA subgrouping was based on barrier variables, sexual activity and sociodemographic correlates, and HIV positivity. | Line 1-3 |
| 5 | 3. There is nothing in the paper that indicated “undiagnosed HIV”. This is a complex concept to measure. | We have removed all instances of this term throughout the manuscript. |  |
| 6 | Abstract. Page 1, lines 35-39: The introduction was incongruous with the rest of the paper. | We have revised the abstract introduction to align with the rest of the paper. | Line 34-39 |
| 7 | Abstract: 5. Page 1, lines 37-39” “….and how these intersections impact patterns of sexual behavior and the prevalence of undiagnosed HIV among adolescents and young people in Nigeria.”: The study did not provide any specific evidence on how the intersections influenced sexual behavior or the prevalence of undiagnosed HIV among adolescents and young people in Nigeria. Additionally, factors related to sexual behavior were considered as potential confounding variables in the association between the latent classes of HIV testing barriers (primary outcome variable) and sociodemographic variables (independent factors). The objective of the study is unclear and needs to be rephrased. | We have now explicitly stated and clarified the objectives in the abstract and throughout the manuscript. We restate the objectives below:    “Our study aims to achieve three objectives:  first, to identify latent classes or subgroups of AYA based on shared patterns of barriers to HIV testing services using LCA.  Second, to identify and compare the sexual and sociodemographic characteristics of AYA across identified latent class or subgroups using LCA regression with covariates.  Third, to explore the association between latent class membership of AYA and observed HIV infection rates using LCA regression with distal outcomes, adjusting for sexual and sociodemographic characteristics”. | Line 36-39, 130-135 |
| 8 | Abstract 6. Page 1, lines 40-43: The authors should clearly specify the methods employed to tackle both the primary and secondary objectives, as well as define the primary and secondary outcome variables. Based on the main findings, it seems that the authors utilized latent clusters as the primary outcome measure, and HIV status as the secondary outcome variable. | We have now explicitly stated and clarified the objectives and the corresponding methods, which we restate below:  “Our study aims to achieve three objectives:  first, to identify latent classes or subgroups of AYA based on shared patterns of barriers to HIV testing services using LCA.  Second, to identify and compare the sexual and sociodemographic characteristics of AYA across identified latent class or subgroups using LCA regression with covariates.  Third, to explore the association between latent class membership of AYA and observed HIV infection rates using LCA regression with distal outcomes, adjusting for sexual and sociodemographic characteristics”.  Also, in the methods, we detailed the statistical analytical methods corresponding to each objective. | Line 130-135  Line 218-259 |
| 9 | Absract7. Page 1, line 43: The term "HIV prevalence" is misleading in this context. It appears that the authors were actually referring to the HIV status determined from the blood samples collected. | We have reframed all instances of HIV prevalence to either HIV status or HIV positive as applicable. |  |
| 10 | Absract8. Page 1, line 44: I would suggest renaming the clusters to improve clarity. Here are my suggestions: Cluster 1: Low-risk perception cluster, cluster 2: Consent and proximity cluster, cluster 3: Testing site cluster and cluster 4: Cost and logistics cluster. | Thank you for the useful terms. We have adopted the reviewer’s suggestions and renamed the classes accordingly. |  |
| 11 | Abstract9. Page 1, line 46: Regarding HIV, it is important to clarify that the percentage of positive test results represents the HIV positivity rate, rather than prevalence. The term 'prevalence' encompasses a broader scope of HIV infection within a particular population." Recall that the study was conducted among AYA who had never been tested for HIV in Nigeria, so prevalence is not applicable. | We have reframed all instances of ‘HIV prevalence’ to either HIV status or HIV positive as appropriate throughout the manuscript. |  |
| 12 | Abstract10. Page 1, line 53: The conclusion does not correspond with the findings. | We have refined to conclusion to accurately reflect the rest of the sections. | Line 56-59 |
| 13 | Introduction: 11. Page 2: The introduction is notably deficient in summarizing the existing body of literature regarding the identifiable factors that impact desired outcomes, particularly in relation to the barriers affecting HIV testing and HIV susceptibility among adolescents and young individuals. | We have rewritten the introduction to reflect the literature on identifiable factors that impact HIV testing barriers and outcomes including HIV vulnerability in AYA. | Line 85-100 |
| 14 | 12. Page 3, line 116: The second question asked about the influence of barriers to HIV testing on sociodemographic characteristics and sexual behaviors. However, the intended question should have been about the impact of sociodemographic characteristics and sexual behaviors on different clusters of barriers to HIV testing. Here is the revised question: "What sociodemographic and sexual behaviors influence different clusters of barriers to HIV testing?" | We have now explicitly stated and clarified the objectives, which we restate below:  “Our study aims to achieve three objectives:  first, to identify latent classes or subgroups of AYA based on shared patterns of barriers to HIV testing services using LCA.  Second, to identify and compare the sexual and sociodemographic characteristics of AYA across identified latent class or subgroups using LCA regression with covariates.  Third, to explore the association between latent class membership of AYA and observed HIV infection rates using LCA regression with distal outcomes, adjusting for sexual and sociodemographic characteristics”. | Line 130-135  Line 218-259 |
| 15 | 13. Page 3, line 118: The third question appears to be unusual. It assessed the relationship between the clusters of barriers to testing and “undiagnosed HIV”. How can one determine undiagnosed HIV? In the methods section, it was mentioned that the participants had never undergone HIV testing. This suggests that individuals who tested positive contributed to the overall HIV positivity rate. Since the analysis is conducted at the individual level, it would be more appropriate to refer the outcome as HIV-positive. | We thank the reviewer for their constructive comments. Accordingly, we have reframed all instances of ‘undiagnosed HIV’ to HIV positivity or HIV status as applicable throughout the manuscript. |  |
| 16 | Methods: 14. Page 3, line 124: “Despite economic growth in recent decades, the country continues to face high poverty rates.” What is the method used to measure the poverty rate? Please provide a reference for the regional poverty rates. | We have included the relevant citation. | Line 155-160 |
| 17 | Page 3, line 154: “According to the national algorithm”: The national algorithm should be stated. | The national algorithm has been referenced and summarised as Supporting Information S1. | Line196 and Supporting Information S1 |
| 18 | Page 4, lines 164-194: It is challenging to determine which variables served as the independent and dependent variables. In contrast to what was stated as the outcome measure in line 194, the primary outcome measure was the barrier to HIV testing, while the secondary outcome measure was HIV status. It is important to state that barrier to HIV testing was selected as an independent variable for the secondary outcome analysis. | We appreciate the reviewer’s constructive comment.  Accordingly, we have clarified objectives. Specifically, the objective now reads:  “Our study aims to achieve three objectives:  first, to identify latent classes or subgroups of AYA based on shared patterns of barriers to HIV testing services using LCA.  Second, to identify and compare the sexual and sociodemographic characteristics of AYA across identified latent classes or subgroups using LCA regression with covariates.  Third, to explore the association between latent class membership of AYA and observed HIV infection rates using LCA regression with distal outcomes, adjusting for sexual and sociodemographic characteristics”.  Additionally, we have refrained from using the terms 'primary' and 'secondary' outcomes because LCA studies typically focus on one outcome – the distal outcome – which in our case is the HIV positivity rate. We believe the framing of our objectives should help resolve any confusion regarding the independent and dependent variables in the study. | Line 130-135  Line 218-259 |
| 19 | 17. There is redundancy from lines 170-194 because the same information was presented in Table 1. | Thank you. We have removed the redundant section. |  |
| 20 | 18. Table 1 should be summarized for clarity’s sake. | We have improved the format and sub-headers to improve readability of the table. | Line 213 |
| 21 | 19. Table 1: The authors should note that gender and sex are not the same. While sex is a biological construct (male and female), gender is a social construct. Based on the question asked, sex is more appropriate. | We have taken this into account and changed all instances of gender to sex throughout the manuscript. |  |
| 22 | 20. Page 6, lines 202-238: Describe the statistical approaches in relation to the objectives. In addition, specify upfront that LCA was employed to reduce data dimensionality of barriers to HIV testing, as its mention arrived quite late in line 220. Also, what type of regression model was performed for HIV status? | We have detailed the statistical approaches in relation the study objectives. Please see Statistical analysis sub-section under Methods, lines 216-259. | Line 216-259 |
| 23 | Results: 21. Table 2 is not clear. Which estimates are frequencies (small n) and medians? | We have corrected this. The authors have chosen to relocate the descriptive tables (Tables 2 and 5) to the Supporting Information. This decision aims to emphasize to readers only Tables with analytical results that directly address the study objectives. | Supporting Information S2 |
| 24 | 22. How is final HIV status in Table 2 operationalized? Also, why is final HIV status placed under sexual activity? | We appreciate the reviewer’s attention to detail. We have corrected this wrong placement.  In addition, we have included in current Table 2 and lines 193-199 how the survey operationalised Final HIV status. | Table 2 in line 213.  Lines 193-199 |
| 25 | 23. Page 10, line 315 (Table 5): The value of p=0.000 does not exist. When the p-value is extremely low, statistical software approximates it to zero. In such cases, it is more appropriate to report the p-value as p<0.001. | We have corrected this wrong usage throughout the manuscript. |  |
| 26 | 24. Table 5: What is the reason behind certain cells being displayed in bold font? | We have removed the bold fonts from this table. See Supporting Information S4. |  |
| 27 | 25. Page 12, line 331: Multinomial regression can not determine association between latent classes. It is used to determine association between independent variables and outcome variable with more than two categories. | Thank you. We have corrected this. |  |
| 28 | 26. Page 13: Table 6: Please indicate what the reported magnitude of effect represents. Are they relative risk ratios (RRR) with 95%CI? | We have indicted the Odds ratios as the measure of effect magnitude in the relevant tables. |  |
| 29 | 27. Page 14, line 373: “(OR 1.68; CI: 1.04-2.71, p =0.04)”: State that this is 95%CI. | We have done this. Thank you. |  |
| 30 | 28. Page 14, line 374: “The association between latent classes and HIV prevalence disappeared”. As stated previously, HIV positivity is more appropriate. | We appreciate the reviewer’s constructive review. We have made this correction throughout the manuscript. |  |
| 31 | 29. Table 7 should reflect crude or unadjusted model. The adjusted model is more important to the reader than Table 7 (unadjusted model). | We appreciate the reviewer’s attention to detail. We have now merged the two tables to show both crude and adjusted OR and 95% CI. Please see current Table 5 on line 405. | Line 405 |
| 32 | Discussion: 30. The identified factors influencing the barriers to HIV testing and HIV infection were not substantially discussed. | We have now substantially discussed the identified factors influencing barriers to HIV testing barriers and HIV infection, including underlying or common determinants of both as applicable to our study. | Lines 430-442, 451-486, |
| 33 | 31. The spatial pattern of clusters of barriers to HIV testing was completed omitted in the discussion section. | We have now integrated the geographical distribution patterns of AYA in the latent classes into our discussion. | Line 454-489 |
| 32 | 32. Page 16, line 447: “The complete profiles (HIV prevalence, socio-demographic factors, and sexual behavior) of the four classes, taken together, offer an understanding of potential pathways through which AYA can transition along the spectrum of sexual risk and HIV vulnerability”: This statement is not accurate because pathways can be identified using LCA. If the authors are interested in identifying pathways, they need to run mediation or structural equation models. | We appreciate the reviewer’s comment. have removed this wording. We have used the word ‘trajectories’ which we believe is more accurate. | Line 492 |
