# Supplementary material for: Latent class analysis of barriers to HIV testing services and associations with sexual behaviour and HIV status among adolescents and young adults in Nigeria
Source: PLoS One. 2024 Apr 18;19(4):e0300220. doi: 10.1371/journal.pone.0300220 (PMC11025812; doi:10.1371/journal.pone.0300220)
Supplement: Attachment — Submitted filename: Second Revision.docx [file pone.0300220.s009.docx]

**General Comments**

References should go before a fullstop at the end of the sentence (i.e., line 70 should read ‘making them accessible to all in need (1,2).’

**Abstract**

*Line 35*: Suggested rewording ‘face multiple barriers to accessing healthcare services, which can interact, creating complex needs that often impact health behaviours’

*Line 40*: Should be ‘data were’ as data is pleural

*Line 43*: Suggested rewording ‘a latent class analysis (LCA) model was built from 12 self-reported variables reporting barrier type’

*Line 46-7*: ‘partner’s HIV status’

*Line 48*: ‘our LCA model identified’

*Lines 50-52*: ‘more likely’ but could you comment on statistical significance? Maybe including a p value or a ratio to indicate the extent of difference?

*Lines 52-53*: suggested rewording ‘there was not significant relationship between barrier subgroup membership and increased HIV infection rates’

*Line 54*: suggested rewording to ‘married/cohabiting’

*Line 57*: change ‘HIV positivity’ to ‘HIV infection’ to match previous terms used

**Introduction**

*Line 73*: Given the readership of Plos One is not specifically HIV focused, it would be worth expanding on what the 95-95-95 targets are.

*Line 81*: move citation to the end of the sentence

*Line 88-89*: give examples of the behavioural issues that you are talking about

*Line 91-92 from ‘linked with’*: this doesn’t make sense, I suggest rewording.

*Line 100-102*: no need for a new paragraph here, I suggest starting new paragraph at ‘to address this need, this study utilises LCA’

*Line 107*: adolescents and young adults should be abbreviated to ‘AYA’

*Line 126-128*: Suggest cutting this sentence as this point has already been discussed a lot

*Line 130*: Suggested rewording to: ‘Our study aims to: identify latent classes or subgroups […], identify and compare the sexual and sociodemographic […], and explore the association […]’

*Line 130*: suggested rewording to ’identify latent classes of AYA’

*Line 133*: remove ‘with covariates’

*Line 135-137*: ‘This study […] Nigeria’ you should move this sentence further up

**Methods**

*Line 157:* Cut ‘as shown in’ and just replace with ‘one Federal capital (*Figure 1*)’

I would suggest moving figure 1 to line 168 (i.e., below the paragraph on educational attainment) as otherwise it splits up the text a bit too much

*Line 172-174*: how were data on HIV/HBV collected? Was it biological sampling or self-reported? It is mentioned below but think it might be worth mentioning something to do with blood tests or biological sampling here, or restructuring this section to put these two points together.

*Line 177-182*: just a suggestion, but might only need to include the response rates and blood draw response rates for the relevant age category of the study (i.e., no need for children aged 0-9)

*Line 183*: The first what?

*Line 184-185*: Eligibility criteria should come earlier in this paragraph

*Line 187*-190*:* No need to say ‘**individual** adult/adolescent questionnaire’ as it becomes quite repetitive, just ‘adult/adolescent questionnaire’ is fine

You need to reference Table 1 in the text as it is currently not referenced anywhere.

*Table 1*: Uncapitalised the question for sex. For the ‘Responses to ‘other’ were re-examined’ in the barriers to HIV testing section, what I assume this means is that if a participant selected other, they were able to offer a free-text response option, which was then analysed by data collectors/your team and assigned an existing category? I think you should add a little more detail on how this was done in the table.

*Line 218*: Remove ‘aligned with our objectives

*Line 243*: space after brackets between functionality and allowed

*Line 245*: replace ‘following the following steps’ with ‘as follows’

*Line 246*: no fullstops after classes before (c)

*Line 259*: Add something here that statistical significance is determined by adjusted ORs not overlapping 1.00

**Results**

*Line 309*: delete ‘as shown in Figure 2’ and just have ‘inclusion criteria (Figure 2)’

*Line 315*: maybe cite a p value when you are saying that it indicated no significant improvement in fit

*Line 324*: replace ‘largest AYA subgroup’ with just ‘largest subgroup’ to avoid the repetition of AYA again

*Line 324-324*: suggest cutting ‘the conditional-response probabilities for barriers […] range from 0.00 to 1.00’. In general don’t think it is needed to quote the range of the conditional response probabilities in my opinion, haven’t seen this done before in papers and adds to the word count.

*Table 3*. Remove ’N=18,612’ from the table title, as it is quite out of place here. Also think you can move the text below up into the title of the table, integrating it into the final sentence about higher conditional response probabilities being bolded.

*Figure 3*. Some more detail in the title of this figure would be welcome, maybe stating that this is a heat map.

*Line 369*: Replace ‘Table 4 shows figures’ with ‘Table 4 shows results’

*Line 370*: justify the use of the low risk-perception class as the reference category (assumably because it is the biggest class)

*Lines 370-372*: include p-values here, and also just more data using Table 4 (i.e., compared to AYA in the low-risk perception class, AYA in the consent and proximity class were 80% more likely to reside in the Northwest (p < 0.05) and 29% less likely to be married (p < 0.05))

*Line 381*: be consistent with tenses, you have used ‘are’ at the end of line 181 but previously have been using ‘were’/‘was’, I recommend going back through the manuscript to check this

*Line 384:* were all three other classes used as reference categories individually? This sentence would benefit from clarification

*Table 4*: in the title, include the acronym for odds ratio (i.e., the reference category for odds ratio (OR)). Also clarify that bolded values indicate statistical significance, as determined by 95% CIs not overlapping 1. In Column 1, make sure all text is bolded in the condom use and number of sexual partners sections. Also, move the ‘OR (95% CI)’ row up into the grey row next to sexual activity. Also need to bold sex.

*Line 401*: clarify what was adjusted for

*Line 404*: include the OR and 95% CI here

*Table 5*: please include p values for all categories, currently none for the sociodemographic characteristics section. Also for reference categories put 1.00 to keep the decimal points consistent. Also why is it 20-25 in the age category of this table, I thought the analysis only included 16-24? Please check these for all the tables and supplementary material. Also explain that the bolded values indicate statistical significance as determined by 95% CIs not overlapping 1.

**Discussion** *Line 426*: is it the first study that does this using nationally representative data, or the first in Nigeria that does so using nationally representative data? Both admirable, but very different points.

*Line 441*: replace with ‘geographical and economic’

*LIne 443-445:* any citations for instances in which LCA has been able to inform policy/other LCAs that are policy focused?

*Lines 446-450*: wondering if you could rephrase some of this to avoid labelling some of these sexual activities as ‘risky’. There has been a recent move against this to incorporate less stigmatising and more sex-positive language. I have attached an article below which could help with reforming the language in this passage while maintaining the points of the paragraph This also applies to the rest of the language of the paper. Marcus JL, Snowden JM. Words Matter: Putting an End to "Unsafe" and "Risky" Sex. Sex Transm Dis. 2020 Jan;47(1):1-3. doi: 10.1097/OLQ.0000000000001065. PMID: 31517770; PMCID: PMC6953392.

*Line 455*: replace ‘could be the common driver’ with ‘could be a common driver’

*Line 459*: replace ‘group within the Northwest region is explained by’ with ‘within the northwest region could be explained by’

*Line 467*: replace sexual reproductive health rights with SRHR as you have already used this acronym

*Line 469*: in the “testing site” *subgroup*

*Line 471*: the prominence *of this subgroup*

*Line 481*: Change the ‘considerable sex risk-taking’ in line with earlier comments about stigmatising language

*Lines 511-526*: please include that trans and gender expansive individuals have been excluded from this analysis because of NAIIS only recording male or female gender, as this is a very strong limitation of the paper that must be addressed in the discussion (note, trans and gender expansive populations are not sexual minority groups, so you need a separate point about this).
