# Supplementary material for: Latent class analysis of barriers to HIV testing services and associations with sexual behaviour and HIV status among adolescents and young adults in Nigeria
Source: PLoS One. 2024 Apr 18;19(4):e0300220. doi: 10.1371/journal.pone.0300220 (PMC11025812; doi:10.1371/journal.pone.0300220)
Supplement: Attachment — Submitted filename: Response to reviewers.docx [file pone.0300220.s010.docx]

Dear Dr. Belayneh Mengist,

Editor,

PLOS ONE

23 November 2023

Resubmission of manuscript “*Latent class analysis of barriers to HIV testing services and associations with sexual behaviour and HIV status among adolescents and young adults in Nigeria”*

Dear Dr Mengist,

We are pleased to submit our revised manuscript entitled: “*Latent class analysis of barriers to HIV testing services and associations with sexual behaviour and HIV status among adolescents and young adults in Nigeria”* to the PLOS ONE for consideration for publication. We appreciate the careful review and constructive suggestions.

We believe that the manuscript is substantially improved after addressing editor and reviewers’ comments. Submitted with this letter are the editor and reviewers’ comments with our responses in tabular form, including how and where the text was modified.

The revision has been developed in consultation with all co-authors, and each author has seen and approved the final form of this revision. The funders had no role in study design, data collection and analysis, decision to publish, or preparation of the manuscript.

Thank you for your consideration of our revised paper.

Sincerely,

Okikiolu Badejo for the co-authors

|  | Editor’s comment | Authors’ response | Location in revised manuscript |
| --- | --- | --- | --- |
|  | Reviewer 1 | Authors’ response | Location in revised manuscript |
| 1 | References should go before a fullstop at the end of the sentence (i.e., line 70 should read ‘making them accessible to all in need (1,2).’ | We thank the reviewer for their attention to detail. We have corrected this. | Line 73 |
| 2 | *Line 35*: Suggested rewording ‘face multiple barriers to accessing healthcare services, which can interact, creating complex needs that often impact health behaviours’ | Done, thank you | Line 35 |
|  | *Line 40*: Should be ‘data were’ as data is pleural | This has been corrected | Line 39 |
| 3 | *Line 43*: Suggested rewording ‘a latent class analysis (LCA) model was built from 12 self-reported variables reporting barrier type’ | We have adopted the suggested rewording | Line 41 |
| 4 | *Line 46-7*: ‘partner’s HIV status’ | This has been corrected | Line 46 |
| 5 | *Line 48*: ‘our LCA model identified’ | Done | Line47 |
| 6 | *Lines 50-52*: ‘more likely’ but could you comment on statistical significance? Maybe including a p value or a ratio to indicate the extent of difference? | Thank you. We have now included odd rations and a95% CI to reflect the extent of difference | Line 51-55 |
| 7 | *Lines 52-53*: suggested rewording ‘there was not significant relationship between barrier subgroup membership and increased HIV infection rates’ | We have adopted the suggested rewording | Line 55-56 |
| 8 | *Line 54*: suggested rewording to ‘married/cohabiting’ | We have adopted the suggested rewording | Line 57 |
| 9 | *Line 57*: change ‘HIV positivity’ to ‘HIV infection’ to match previous terms used | As per other reviewer’s comment, we have replaced all uses of HIV infection or HIV infection rates to HIV status | Line 60 |
| 10 | *Line 73*: Given the readership of Plos One is not specifically HIV focused, it would be worth expanding on what the 95-95-95 targets are. | Thank you, this has been done | Line 76-78 |
| **11** | *Line 81*: move citation to the end of the sentence | Done | Line 89 |
| 12 | *Line 88-89*: give examples of the behavioural issues that you are talking about | We have rephrased this passage to read “These barriers include financial, social (such as stigma), and informational obstacles” | Line 95-96 |
| 13 | *Line 91-92 from ‘linked with’*: this doesn’t make sense, I suggest rewording. | We have reworded this to read “In Nigeria, there is a noticeable lack of services related to mental health, reproductive health, clinic transitional care, and psychosocial support for young people living with HIV (15) which might explain the poor health-seeking behaviour and heightened vulnerability to HIV observed in this age group” | Line 96-96 |
| 14 | *Line 100-102*: no need for a new paragraph here, I suggest starting new paragraph at ‘to address this need, this study utilises LCA’ | Thank you. We have done this | Line 112 |
| 15 | *Line 107*: adolescents and young adults should be abbreviated to ‘AYA’ | Done | Line113 |
| 16 | *Line 126-128*: Suggest cutting this sentence as this point has already been discussed a lot | Done |  |
| 17 | *Line 130*: Suggested rewording to: ‘Our study aims to: identify latent classes or subgroups […], identify and compare the sexual and sociodemographic […], and explore the association […]’ | We have adopted the suggested rewording | Line 136-140 |
| 18 | *Line 130*: suggested rewording to ’identify latent classes of AYA’ | Done | Line 136 |
| 19 | *Line 133*: remove ‘with covariates’ | Done |  |
| 20 | *Line 135-137*: ‘This study […] Nigeria’ you should move this sentence further up | Done | Line 131-133 |
| 21 | *Line 157:* Cut ‘as shown in’ and just replace with ‘one Federal capital (*Figure 1*)’ | Done | Line 161 |
| 22 | I would suggest moving figure 1 to line 168 (i.e., below the paragraph on educational attainment) as otherwise it splits up the text a bit too much | Done |  |
| 23 | *Line 172-174*: how were data on HIV/HBV collected? Was it biological sampling or self-reported? It is mentioned below but think it might be worth mentioning something to do with blood tests or biological sampling here, or restructuring this section to put these two points together. | Thank you. We have restructured the section to bring the two points together | Line 182-189 |
| 24 | *Line 177-182*: just a suggestion, but might only need to include the response rates and blood draw response rates for the relevant age category of the study (i.e., no need for children aged 0-9) | Done, we have removed the non-relevant age category |  |
| 25 | *Line 183*: The first what? | We have edited the passage to read “To our knowledge, this is the first study to explore patterns of barriers to HIV testing services among AYA in the context of sexual and sociodemographic characteristics and HIV status…” | Line 188 |
| 26 | *Line 184-185*: Eligibility criteria should come earlier in this paragraph | We have moved eligibility criteria under the first paragraph under Data sub-section of the Method | Line 178-180 |
| 27 | *Line 187*-190*:* No need to say ‘**individual** adult/adolescent questionnaire’ as it becomes quite repetitive, just ‘adult/adolescent questionnaire’ is fine | Done |  |
| 28 | You need to reference Table 1 in the text as it is currently not referenced anywhere. | Done | Line 214 |
| 29 | *Table 1*: Uncapitalised the question for sex. For the ‘Responses to ‘other’ were re-examined’ in the barriers to HIV testing section, what I assume this means is that if a participant selected other, they were able to offer a free-text response option, which was then analysed by data collectors/your team and assigned an existing category? I think you should add a little more detail on how this was done in the table. | Thank you. We have uncapitalised the question for sex. We have rephrased the ‘other’ response which now reads ‘Responses to ‘Other’ were provided as free-text. NAIIS survey team analysed the free-text responses and assigned each back into existing categories.’ | Line 216 |
| 30 | *Line 218*: Remove ‘aligned with our objectives | Done |  |
| 31 | *Line 243*: space after brackets between functionality and allowed | Done |  |
| 32 | *Line 245*: replace ‘following the following steps’ with ‘as follows’ | Done | Line 247 |
| 32 | *Line 246*: no fullstops after classes before (c) | Done |  |
| 34 | *Line 259*: Add something here that statistical significance is determined by adjusted ORs not overlapping 1.00 | Done | Line 261-263 |
| 35 | *Line 309*: delete ‘as shown in Figure 2’ and just have ‘inclusion criteria (Figure 2)’ | Done | Line 314 |
| 36 | *Line 315*: maybe cite a p value when you are saying that it indicated no significant improvement in fit | Done | Line 320 |
| 37 | *Line 324*: replace ‘largest AYA subgroup’ with just ‘largest subgroup’ to avoid the repetition of AYA again | Done |  |
| 38 | *Line 324-324*: suggest cutting ‘the conditional-response probabilities for barriers […] range from 0.00 to 1.00’. In general don’t think it is needed to quote the range of the conditional response probabilities in my opinion, haven’t seen this done before in papers and adds to the word count. | Done |  |
| 39 | *Table 3*. Remove ’N=18,612’ from the table title, as it is quite out of place here. Also think you can move the text below up into the title of the table, integrating it into the final sentence about higher conditional response probabilities being bolded. | Done | Line 339 |
| 40 | *Figure 3*. Some more detail in the title of this figure would be welcome, maybe stating that this is a heat map. | We have now stated the title to read “Spatial map showing zonal geographical distribution of AYA by latent class subgroups‘’ | Line 371 |
| 41 | *Line 369*: Replace ‘Table 4 shows figures’ with ‘Table 4 shows results’ | Done | Line 374 |
| 42 | *Line 370*: justify the use of the low risk-perception class as the reference category (assumably because it is the biggest class) | Done | Line 375 |
| 43 | *Lines 370-372*: include p-values here, and also just more data using Table 4 (i.e., compared to AYA in the low-risk perception class, AYA in the consent and proximity class were 80% more likely to reside in the Northwest (p < 0.05) and 29% less likely to be married (p < 0.05)) | We have included odd ratios with corresponding 95% CI in all the relevant places of text | Line 377-398 |
| 44 | *Line 381*: be consistent with tenses, you have used ‘are’ at the end of line 181 but previously have been using ‘were’/‘was’, I recommend going back through the manuscript to check this | Thank you. We have now kept all tenses consistent throughout the manuscript |  |
| 45 | *Line 384:* were all three other classes used as reference categories individually? This sentence would benefit from clarification | Done. The sentence now reads “To determine the profile of AYA in the low-risk perception class, we reset the reference category by using each of the three other classes individually as reference categories in our analysis ” | Line 399-400 |
| 46 | *Table 4*: in the title, include the acronym for odds ratio (i.e., the reference category for odds ratio (OR)). Also clarify that bolded values indicate statistical significance, as determined by 95% CIs not overlapping 1. In Column 1, make sure all text is bolded in the condom use and number of sexual partners sections. Also, move the ‘OR (95% CI)’ row up into the grey row next to sexual activity. Also need to bold sex. | Thank you for your attention to detail. For this table, we have now clarified what bolded values mean. To avoid confusion for the readers, we have reserved bolding for only values that indicate statistical significance. No other text in the table appears in bold anymore | Line 410 |
| 47 | *Line 401*: clarify what was adjusted for | Done | Line 416-417 |
| 48 | *Line 404*: include the OR and 95% CI here | Done | Line 417 |
| 49 | *Table 5*: please include p values for all categories, currently none for the sociodemographic characteristics section. Also for reference categories put 1.00 to keep the decimal points consistent. Also why is it 20-25 in the age category of this table, I thought the analysis only included 16-24? Please check these for all the tables and supplementary material. Also explain that the bolded values indicate statistical significance as determined by 95% CIs not overlapping 1. | For Table 5 we have done the following   - Kept decimal points consistent by changing 1 to 1.00 - Corrected age groups to 15-19 and 20-24 (also in supplementary material) - Explained what bolded values mean in the title   To maintain consistency with the other analytic tables (Table 4 and supplementary material) we have dropped p-values presented only OR and 95% CI. |  |
| 50 | *Line 426*: is it the first study that does this using nationally representative data, or the first in Nigeria that does so using nationally representative data? Both admirable, but very different points.  *Line 441*: replace with ‘geographical and economic’ | We have reworded this statement for clarity. It now reads “To our knowledge, this is the first study to explore patterns of barriers to HIV testing services among AYA in the context of sexual and sociodemographic characteristics and HIV status, using a nationally representative data” | Line 422 |
| 51 | *LIne 443-445:* any citations for instances in which LCA has been able to inform policy/other LCAs that are policy focused? | Reference has been included | Line 447 |
| 52 | *Lines 446-450*: wondering if you could rephrase some of this to avoid labelling some of these sexual activities as ‘risky’. There has been a recent move against this to incorporate less stigmatising and more sex-positive language. I have attached an article below which could help with reforming the language in this passage while maintaining the points of the paragraph This also applies to the rest of the language of the paper. Marcus JL, Snowden JM. Words Matter: Putting an End to "Unsafe" and "Risky" Sex. Sex Transm Dis. 2020 Jan;47(1):1-3. doi: 10.1097/OLQ.0000000000001065. PMID: 31517770; PMCID: PMC6953392. | We thank the reviewer for this very important observation and suggestions.  We have rephrased all instances of stigmatising sex-behaviour language following the guidance and suggestion of the paper. This included removing all uses of ‘risk’ or ‘risky’ in the context of sexual behaviour. We have reserved the use of ‘risk’ or ‘risky’ only in the context HIV acquisition, or perception. |  |
| 53 | *Line 455*: replace ‘could be the common driver’ with ‘could be a common driver’ | Done | Line 456 |
| 54 | *Line 459*: replace ‘group within the Northwest region is explained by’ with ‘within the northwest region could be explained by’ | Done | Line 460-462 |
| 55 | *Line 467*: replace sexual reproductive health rights with SRHR as you have already used this acronym | Done | Line 468 |
| 56 | *Line 469*: in the “testing site” *subgroup* | Done | Line 469 |
| 57 | *Line 471*: the prominence *of this subgroup* | Done | Line 472 |
| 58 | *Line 481*: Change the ‘considerable sex risk-taking’ in line with earlier comments about stigmatising language | Done |  |
| 58 | *Lines 511-526*: please include that trans and gender expansive individuals have been excluded from this analysis because of NAIIS only recording male or female gender, as this is a very strong limitation of the paper that must be addressed in the discussion (note, trans and gender expansive populations are not sexual minority groups, so you need a separate point about this). | We have emphasized and made this limitation more explicit. Thank you | Line 520-529 |
|  |  |  |  |
| 1 | The manuscript has undergone significant revisions by the authors, resulting in notable improvements to its quality. Nevertheless, additional revisions are still necessary. | We thank the reviewers for their valuable reviews which has improved the quality of the manuscript. |  |
| 2 | Page 39, line 51: In the abstract and body of the manuscript, the authors utilized the term "HIV infection rate" to describe their objectives and inferential analyses. While the use of rate for descriptive analysis is acceptable, it is not appropriate for inferential statistics as the outcome was not aggregated. HIV status was at the individual level. | We thank the reviewer for this comment. We have changed all uses of HIV infection rates to HIV status throughout the abstract and body of the manuscript. |  |
| 3 | Page 39, line 57: "Data was..." should be revised to "Data were..." | Done. | Line 39 |
| 4 | Page 39, lines 60 and 61: Replace "LCA regressions..." with "Latent Class Regressions (LCR)." Also, make corresponding changes in the body of the manuscript. | Done. | Line 43, 126, |
| 5 | Page 41, lines 113 and 114: “In 2021, the number of new infections among adolescent girls and young women aged 15 to 24 decreased to an estimated 400,000." The authors should state the comparative year and the number of new infections. | Thank you. We have reworked the section to reflect the comparative year, number and percentage decline. | Line 83-85 |
| 6 | Page 42, lines 169–171: The authors’ claim regarding the novelty of their study is inaccurate. Several studies have used LCA to identify barriers to health services.  https://bmchealthservres.biomedcentral.com/articles/10.1186/1472-6963-11-181  https://www.ncbi.nlm.nih.gov/pmc/articles/PMC10412727/ | We thank the reviewer for their attention to detail. We have now edited the section to convey our novelty in terms of the application of LCA and LCR to investigating association between barriers to HIV testing services, sexual behaviour and HIV status in young people, rather than just the application of LCA to investigate barriers to health services. | Line 129-131 |
| 7 | Page 45, line 294: “For children aged 0–9 years, the blood draw response rate was 68.5% for women and men”. Considering the age group, boys and girls are appropriate. | Per the other reviewer’s comment, we have taken out this section of the manuscript. |  |
| 8 | Page 46, line 324: Is there any rationale for limiting the analysis to those who only engage in vaginal sex, considering that young people participate in various high-risk behaviors such as anal sex, injection drug use, etc.? In line 335, it appears that NAIIS used only vaginal sex as a criterion to identify those who were sexually active. Are there any implications of narrowly defining the HIV risk within this population, particularly considering that injection drug use is becoming a public health challenge in Nigeria? | We thank the reviewer for this comment. Accordingly, in our limitation section, we have stated that focusing solely on vaginal sex and sexual activity to explain HIV positivity might have led us to overlook the significant contribution of other non-sexual transmission routes, such as injection drug use. | Lines 524-529 |
| 9 | In the manuscript, there are several occurrences, including Fig. 3, where the authors have utilized the term "prevalence" instead of "positivity." It is important to note that prevalence encompasses both old and new cases, whereas positivity rate specifically refers to the proportion of individuals who tested positive out of the total number of people tested. | Thank you. We have replaced all uses of prevalence with HIV status. |  |
