# Supplementary material for: Latent class analysis of barriers to HIV testing services and associations with sexual behaviour and HIV status among adolescents and young adults in Nigeria
Source: PLoS One. 2024 Apr 18;19(4):e0300220. doi: 10.1371/journal.pone.0300220 (PMC11025812; doi:10.1371/journal.pone.0300220)
Supplement: Attachment — Submitted filename: Response to reviewers_3rd round.docx [file pone.0300220.s011.docx]

Dear Dr. Belayneh Mengist,

Editor,

PLOS ONE

22 January 2024

Resubmission of manuscript “*Latent class analysis of barriers to HIV testing services and associations with sexual behaviour and HIV status among adolescents and young adults in Nigeria”*

Dear Dr Mengist,

We are pleased to submit our revised manuscript entitled: “*Latent class analysis of barriers to HIV testing services and associations with sexual behaviour and HIV status among adolescents and young adults in Nigeria”* to the PLOS ONE for consideration for publication. We appreciate the careful review and constructive suggestions.

We believe that the manuscript is substantially improved after addressing editor and reviewers’ comments. Submitted with this letter are the editor and reviewers’ comments with our responses in tabular form, including how and where the text was modified.

The revision has been developed in consultation with all co-authors, and each author has seen and approved the final form of this revision. The funders had no role in study design, data collection and analysis, decision to publish, or preparation of the manuscript.

Thank you for your consideration of our revised paper.

Sincerely,

Okikiolu Badejo for the co-authors

|  | Editor’s comment | Authors’ response | Location in revised manuscript |
| --- | --- | --- | --- |
| 1 | First, please try to see the comments provided in R2 and address them. For example, you have been advised to use references appropriately (cited after a full stop). See line 75, 76, 81….224, 254….524. | We thank the editoer for their attention to details. We have now consistently applied the citation format throughout the manuscript |  |
| 2 | In the abstract, put Odds Ratio (OR) once; there is no need to put OR repeatedly. The same is true for 95% CI. | Acknowledging the reviewer's comments, the section has been revised to report only significant associations. Rather than batching the findings for the AYA classes, individual statistics for each AYA class, including adjusted odds ratios (aOR) and 95% confidence intervals (CI), have been provided for clarity. | Line 50-60 |
| 3 | You reported simply OR; it is not clear whether COR or AOR were reported. As long as a was regression adjusted for factors, AOR should be reported. |  |  |
| 4 | Findings are reported from non-significant estimates. In line 51: OR 1.14, 95% CI 0.88-1.48, line 54; OR 1.24, 95% CI 0.98-1.57, OR 1.31, 95% CI 0.79-2.15 and line 55; OR 0.82, 95% CI 0.66-1.02. I know that non-significant findings can be reported as no association, but you generalized as having an association. |  |  |
| 5 | Line 234: Citation is needed. | Done | Line 236 |
| 6 | In Table 4, you need to put variables adjusted for in the model as a footnote. | Done |  |
| 7 | When fitting models, multicollinearity should be considered. For example, in your model reported in Table 4, “place of residence” and “zone” might have a potential correlation. Please consider it. | Thank you for your comment. In response to your suggestion, we have conducted and incorporated additional diagnostics into our analyses to assess all predictors in the regression model for potential collinearity. This includes examining and reporting correlation matrices and variance inflation factors (VIFs) to assess for potential colinearity. | Line 260-262  Line 380-385 |
| 8 | It is worthwhile to explain a bit more about how categories/subgroups (“low-risk perception, consent and proximity, testing site and cost and logistics”) were constructed. I recommend to clearly explain this in the method section. | In response to the reviewer's suggestion, we have incorporated a detailed explanation of the construction of latent class subgroups in the method section. | Line 223-247 |
| 9 | In Table 5, the “other” in the religion category is referenced; put as a footnote. | Thank you, we have done this | Line 435-436 |
| 10 | It would be useful to conduct a subgroup analysis across sexes rather than putting it as a limitation. | Thank you for the suggestion. While acknowledging the limitation of not including a sex subgroup analysis, our approach to conducting a multivariable analysis, including sex as a variable, aligns with our aim for a comprehensive understanding of how sociodemographic and sexual factors related to barriers to HIV testing can influence vulnerability to HIV among adolescents and young people. While a sex subgroup analysis can isolate effects within sexes, our multivariate analyses sufficiently examine the collective impact and interplay of sexual and sociodemographic factors, including sex. |  |
|  | Reviewer #1 comment |  |  |
| 11 | Thank you very much for addressing these comments, the manuscript is now substantially improved and I can recommend it for publication | Thank you |  |
| 12 | However, there is just one small change required. Line 522 currently reads 'may exclude the perspective of sexual minorities or key population groups, transgenders, and other gender expansive individuals'. Transgenders is a slightly outdated term to use. Also, the important point I have been making in revisions about trans and gender expansive communities has not necessarily been about the sexual activity of the community as suggested in this most recent revision, but rather is about the exclusion of the group from the NAIIS survey as a whole. I therefore suggest the sentence is replaced with 'Moreover, the use of only vaginal sex as a measure of sexual activity in NAIIS excludes the experiences and activities of sexual minority groups, for example. In addition to this, the binary coding of sex in NAIIS excludes transgender and other gender expansive individuals from our analysis. These are significant communities in the context of the HIV epidemic in Nigeria and other settings (72,73), and future research should focus on sexual behaviours and the current HIV epidemic in these communities.' | Thank you. We have now replaced the section with the suggested statement. | Line 538-544 |
|  | Reviewer #2 comments |  |  |
| 13 | The authors have used multiple imputations to treat missing data. To ensure reliability of their results, the authors should conduct sensitivity analysis and assess concordance or discordance of results from the imputed dataset and original dataset. | Thank you. We have now conducted a sensitivity analysis, incorporating findings from both non-imputed and imputed datasets, mirroring the LCA and LCR with distal outcomes. In our discussion, we carefully considered the implications of both the similarities and differences observed, offering insights into the reliability of our study findings. | Line 272-277  Line348-353  Line 552-565 |
| 14 | Page 47, lines 304-307: The authors wrote odd instead of odds. | We have corrected this throughout the manuscript |  |
| 15 | Page 49, line 370: Table 2: As highlighted in my previous comments, p-value=0.0000 should be rewritten as p-value <0.001. | Done |  |
| 16 | Page 52, The age groupings in other tables are not consistent with table 4, supplementary 2 and supplementary 4. | We have correceted the inconsistencies |  |
| 17 | Supplementary 4; “correlates” was wrongly spelt. | We have corrected this |  |
