# Supplementary material for: Latent class analysis of barriers to HIV testing services and associations with sexual behaviour and HIV status among adolescents and young adults in Nigeria
Source: PLoS One. 2024 Apr 18;19(4):e0300220. doi: 10.1371/journal.pone.0300220 (PMC11025812; doi:10.1371/journal.pone.0300220)
Supplement: Attachment — Submitted filename: Response to reviewers_4th round.docx [file pone.0300220.s012.docx]

Dear Dr. Belayneh Mengist,

Editor,

PLOS ONE

21 February 2024

Resubmission of manuscript “*Latent class analysis of barriers to HIV testing services and associations with sexual behaviour and HIV status among adolescents and young adults in Nigeria”*

Dear Dr Mengist,

We are pleased to submit our revised manuscript entitled: “*Latent class analysis of barriers to HIV testing services and associations with sexual behaviour and HIV status among adolescents and young adults in Nigeria”* to the PLOS ONE for consideration for publication. We appreciate the careful review and constructive suggestions.

We believe that the manuscript is substantially improved after addressing editor and reviewers’ comments. Submitted with this letter are the editor and reviewers’ comments with our responses in tabular form, including how and where the text was modified.

The revision has been developed in consultation with all co-authors, and each author has seen and approved the final form of this revision. The funders had no role in study design, data collection and analysis, decision to publish, or preparation of the manuscript.

Thank you for your consideration of our revised paper.

Sincerely,

Okikiolu Badejo for the co-authors

|  | Editor’s comment | Authors’ response | Location in revised manuscript |
| --- | --- | --- | --- |
| 1 | Thank you for addressing the comments. The manuscript is substantially improved and ready for publication. Some minor editorial comments. | We thank the editors and reviewers for their helpful reviews which improved the quality of the manuscript |  |
| 2 | There is some mixing of the method and result parts; for example, Figure 2 is the method section, and Line 185 to 188 is part of the result section. | Thank you for your comment regarding the placement of Figure 2 within the manuscript. The figure was relocated from its previous location(in the methods) to it current location (in results) based on the recommendation provided by the reviewers during the revision process, which aimed to enhance the clarity, coherence, and logical flow of the manuscript.  Returning Figure 2 to its previous location within the methods section at this stage would not only disrupt the established structure and flow of the manuscript but also potentially confuse readers. Moreover, reverting the change now would consume additional time and resources, which may be better allocated to addressing other critical aspects of the manuscript.  We appreciate your understanding and assure you that careful consideration was given to the placement of Figure 2 to ensure the overall coherence and readability of the manuscript. |  |
|  |  | Thank you for bringing up the concern regarding the classification of Lines 185 to 188 as part of the results section. Upon careful examination, we would like to clarify that the content presented in these lines is not derived from our study's analysis but rather a comprehensive description of the survey utilized to gather the data for our study within Lines 173 to 202. The purpose of Lines 185-188 and broadly 173-202 was to offer readers essential context about the original survey, thereby ensuring transparency and clarity regarding the data source utilized in our analysis.  Moving this information to the results section could potentially mislead readers into believing that it constitutes findings from our study's analysis, which is not the case. Therefore, maintaining the placement of Lines 185 to 188 within the Methods section is crucial to accurately represent the structure of our manuscript and to prevent any confusion among readers regarding the origin of the reported data.  We appreciate your helpful comments to ensure accuracy and clarity of our manuscript for the benefit of the readers. |  |
| 3 | Some minor editorial issues, for example, at line 330 and 435, need space “thefour”, “figuresin” | We have corrected these |  |
| 4 | I recommend explaining the variables adjusted for in the multivariable models as a footnote. | Thank you for your comment to explain the variables adjusted for in the multivariable models. We appreciate the opportunity to address this concern.  We would like to clarify that in our analysis, all variables included in the multivariable models were mutually adjusted for one another. To provide further transparency and ensure clarity for readers, we included a footnote under Tables 4 and 5 stating this adjustment while directing readers to Table 1 for a detailed description of each variable. We hope this is satisfactory and thank you for your very helpful comments. |  |
